# Supplementary material for: Heterogeneity matters: Contact structure and individual variation shape epidemic dynamics
Source: PLoS One. 2021 Jul 20;16(7):e0250050. doi: 10.1371/journal.pone.0250050 (PMC8291658; doi:10.1371/journal.pone.0250050)
Supplement: Attachment — Submitted filename: report.pdf [file pone.0250050.s001.pdf]

Report for PONE-D-21-09416

In the manuscript "Why ODE models for COVID-19 fail: Heterogeneity shapes epidemic dynamics", `PONE-D-21-09416`, the authors investigate how variations in the connectivity and infectivity of individuals affects the evolution of pandemics and in particular features such as the evolution of the effective reproduction number (R_t), the total number of infections, and the heard immunity threshold (HIT). To this end, the authors investigate the evolution of pandemics on networks with varying connectivity characteristics and with either constant or individually varying infection rates and compare these with results obtained by a model using ordinary differential equations (ODE) with the same compartments. To establish a meaningful comparison, either between different networks or between the network-based approaches and the ODE model, the authors choose their parameters according to certain conditions, which in general require that the basic reproduction number (R_0) is equal between all variations of the models. The experiments presented reveal that variations in connectivity and the distribution of infection rates among individuals affects the evolution of the pandemic as measured by the time evolution of a number of quantities of interest defined in the manuscript, and in particular varies from the ODE model.

I find the methodology used by the authors to be sound, the analysis thorough, and the manuscript well written and clear. The results are well presented and the figures are discussed adequately. However, before I can recommend publication, I would suggest clarifying some points I have identified, or alternatively revise the manuscript as explained in the following.

Major points

• A main issue I see is related to the authors' conclusion on the inadequacy of ODE models in modelling COVID-19. In fact, the strongest point made by the authors with respect to this is in the title, which suggests that ODE models "fail". In the manuscript on the other hand, the conclusion drawn is not as broad: that ODE models, in their simplest form, exhibit tension with network-based approaches when the latter include heterogeneity and when the model parameters between them are set to yield the same R_0 or average infection rate. In many cases however, for ODE models, parameters are obtained from data, e.g. via fitting, while other examples include ODE approaches with time-dependent infection rates. ODE models still play an important role to extract, e.g. R_t from data and evaluate its time-dependence. I therefore believe that the authors should support the broad statement made in the title, i.e. that ODE models fail, by showing or explaining why they believe, for example, that their conclusions apply when additional approaches are used in fixing the parameters between the models, including data driven approaches. Otherwise I suggest the title be revised to reflect more accurately the conclusion.

• According to the manuscript, the authors use a fixed number of nodes (N) throughout, namely $N = 1000$. Given that ODE approaches relate the compartments as fractions and are therefore independent of the population size, the

authors should comment on the choice of N and should argue how their conclusions may vary with this choice. For example, can the discrepancy between the ODE result and the fully connected, homogeneous network be an indication of the error made by the choice of N, given that the two should coincide precisely when $N \to \infty$? The authors should clarify this in the text.

• On lines 376 - 378, the authors state: *"Regarding the dispersion, we see that no network structure by itself leads to a dispersion where 80% of the infections are caused by only 15% of the infected nodes [19] (at least initially)"*. However, if I read Figs. 7 or 8 correctly, for the power-law case, one can see that 80% of the infected are from between 10 - 20% of the infected nodes after the tenth day or so, which, when one allows for some uncertainty, is not inconsistent with the conclusion of Ref. [19]. The authors should clarify this statement in light of these figures. For example, the authors could plot the 15% case in addition to the 10%, 20%, and 30% cases in Figs. 7 and 8 or alternatively could comment on the magnitude of the stochastic errors of the points.

Minor suggestions

I also have a number of minor suggestions which I believe can help improve the presentation:

• On line 52, the authors should qualify *"weaken the epidemic"*. For example, lower HIT, lower peak infection, etc.

• On line 56, I believe it is difficult for the reader to understand what *"imposing common interaction structures"* means at this point in the manuscript. I would suggest the authors rephrase.

• In section "Related work", it is not clear why the authors discuss branching processes. The authors state: *"We focus on the three model types, we consider most relevant for epidemiological modeling in general"*. While it is unrealistic to expect the authors to review all types of models available, including e.g. particle based approaches or lattice-based models, the choice of introducing branching processes, in addition to the ODE and network-based approaches which are used to obtain results in this work, is confusing. The authors should either be clearer in the text on why they choose to discuss branching processes here or restrict to introducing the two approaches used for the results that follow. Item 1. of the enumerated contributions on lines with numbers 54 and 55 should also be revised accordingly.

• The authors should give the relation that yields γ from R_0. I understand that this is a reworking of the similar (unnumbered) relation in the supplementary material of Ref. [71], but it would improve the completeness of the presentation if the relation is given around line 178 using the notation of this manuscript.

• On lines 212 to 214 the authors state: *"We define R_0^i as the basic reproduction number when the infection starts in node i and find that $R_0 = E[R_0^i]$"*, however

this appears to be a *definition* of R_0 in this case. The authors should either change the text to clarify that this is the definition of R_0, or should alternatively provide the definition of R_0. Furthermore, it should be clarified in this context how $E[R_0^i]$ is computed, i.e. presumably this is the unweighted average.

• In Fig. 4, the complete graph is not presented, however the placement of the reference to Fig. 4 seems to imply this. I would suggest the authors either include an example of a complete graph in Fig. 4 for completeness, or alternatively move the reference to Fig. 4 to around line 230 where the networks that are depicted are introduced. I would also suggest the networks in Fig. 4 occur in the order they are presented in the text.

• I would suggest the authors explicitly give the form of ν, preferably around line 220, as a function of λ_i and λ.

• On line 280, the authors should quantify *"high accuracy"*. For example, was the number of simulation runs determined by demanding an upper limit for the uncertainty in the compartment fractions at some given time?

• On line 351, the authors write: *"Varying infectiousness generally weakens the epidemic's impact."*. I believe this statement may be misunderstood by the reader and rather than using the word "varying" the sentence would be clearer if rephrased. For example: *"Allowing infectiousness to vary between individuals generally weakens the epidemic's impact."*

• The same as above on line 358 concerning the use of the word *"Varying"*. On the same line, the authors should qualify what they refer to as *"stochasticity"*. For example, do they mean the variance in R_t, i.e. the magnitude of CoV?

• Typos:
  - Starting on line 75: *"We focus on the three model types, we consider ..."* → *"We focus on the three model types we consider ..."* i.e. comma is misplaced
  - Line 98: *"... applications a summarized ..."* → *"... applications are summarized ..."*
  - Caption of Fig. 2: *"... disease sage is ..."* → *"... disease stage is ..."*
  - Line 185: *"... susceptible top exposed ..."* → *"... susceptible to exposed ..."*
  - Line 205: *"... p_I is the conceptually ..."* → *"... p_I is conceptually ..."*
  - Line 208: *"Like in ODE-approach ..."* → *"Like in the ODE-approach ..."*
  - Line 267: *"Thereby, ensure that ..."* → *"Thereby, ensuring that ..."*
  - Line 294: Missing section name after *"Section"*
  - Line 330: *"expextable"* → *"expected"*
