# Supplementary material for: Heterogeneity matters: Contact structure and individual variation shape epidemic dynamics
Source: PLoS One. 2021 Jul 20;16(7):e0250050. doi: 10.1371/journal.pone.0250050 (PMC8291658; doi:10.1371/journal.pone.0250050)
Supplement: Attachment — Submitted filename: response_to_reviewers.pdf [file pone.0250050.s002.pdf]

Response to Reviewers

Dear Reviewers,

We thank you for your very constructive and detailed reviews, which contain a lot of valuable suggestions to improve the presentation and more clearly explain the method and the results.

Most importantly, considering that both of you raised concerns regarding the title of the manuscript, we decided to change it to:

"Heterogeneity matters: Contact structure and individual variation shape epidemic dynamics"

We hope that this better reflects the analysis and the results of our study.

Next, we answer specific concerns raised. For clarity, small issues like typos/formulations are updated without explicitly mentioning them here. Line numbers are based on the file *Revised Manuscript with Track Changes*.

Review 1

1) The title of the manuscript seems misleading to me since the authors make use of a translated ODE model to investigate different contact networks and study COVID-19 spread. It seems that the authors may have intended to mention that the ODE models alone are incomplete to describe the COVID-19 spread. I suggest that the authors make a brief comment about this. In the present version, it sounds like ODE models are useless.

We adjusted the title of the manuscript.

2) The authors incorporate in their analysis the so-called "super-spreaders". What about the "super non-spreaders", i.e., people who have dramatically reduced contacts with others? This may be the case for most elderly people. Does the employed model take this into account? This may be related to age, since young people contribute to a higher number of infections than older people, see, for instance, Science 371, 1336 (2021). I consider that this point deserves a brief discussion in the manuscript.

Like with super-spreaders, *"super non-spreaders"* might fall into two categories:

1. Individuals that drastically reduce their contacts and
2. Individuals that exhibit very low viral load levels (or a combination of those two).

We observe the former in the power-law contact networks as there are many nodes with a very small number of neighbors (one or two). We study the latter in **Experiment 2** where, by using an exponentially distributed infectiousness, many nodes infect their neighbors with a very small probability.
For over-dispersed epidemics, like COVID-19, most nodes are actually *"super non-spreaders".* We clarified this in the manuscript (line 268):

> *Note that overdispersion inevitably indicates not only the existence of super-spreaders but also the existence of nodes that are unlikely to pass the infection at all. Like super-spreaders, these individuals might be the result of host properties (i.e., a low viral load) or connectivity differences.*

3) In line 168, the authors write:
"... In contrast to [71], we merge dead and recovered stages to a single removed stage, as both do not influence the infection dynamics further (we assume immunity after recovery). ..."
The authors should be careful in stating that recovered people do not further influence the infection dynamics. Recovered people, as the authors assume, are immune to some extent (not considering, for instance, distinct variants of the virus) and can act as suppressors of the creation of new infection branches in contact networks.

This is an important point and we clarified in the manuscript that this assumption does not hold (line 180):

> *Note that perfect and permanent immunity is not given for COVID-19. In this study, we ignore the impact of re-infected individuals.*

Merging *dead* and *recovered* only makes sense given the premise of the original model where recovered nodes are assumed to be completely immune. Making this disease progression more realistic and allowing re-infections of recovered (and vaccinated) individuals would be another interesting research direction but we believe that it is not particularly relevant for the aspects we analyze in this study.

4) Why do the authors have specifically employed a SIR-type approach on the ODE model (line 165) instead of, for instance, a SEIS- or a SEIRS-type model? This question is connected with the point raised in 3).

While SEIS-type models, where recovered individuals lose their immunity after some time, are very interesting theoretically and certainly useful in some epidemic contexts, we find that most models in literature on COVID-19 are a variant of the SIR paradigm.
We believe that the reason for that is that most scientists seem to be convinced that re-infected individuals are not a significant (or even noticeable) driver of the epidemic. Naturally, this might

change within this year. Moreover, data on re-infection probabilities, especially w.r.t. novel variants of SARS-CoV-2, were very weak at the time when we conducted the study.
Finally, our goal was not to find the most realistic COVID-19 model but one that allows us to study the effects of population heterogeneity in a principled manner. Thus, we chose a model from literature that captures essential properties of COVID-19 (like asymptomatic infections) but where we simplify enough to keep the research question in focus.
Moreover, SIS-type models are typically analysed in the equilibrium phase while here we focus on effects of heterogeneity in the transient regime.

5) In the caption of Fig.7, the authors write:
"... note a significant amount of noise in the power-law case). ..."
Such noise in the power-law case can also be observed in Fig.8. What is the origin of such a noise? What does this noise mean and why it is not observed for the other cases? I suggest that the authors comment on this aspect.

The noise is the natural result of stochastic fluctuations. The reason for this is that almost all trajectories (individual simulations) die out after about 20 time units. This is not the case for the other networks because their die-out probability is not as high. For example, in the complete graph, most trajectories die out after 100 time units but, still, enough samples remain for a good estimation of the corresponding summary statistics.
We clarified this in the manuscript (line 322):

> *Note that measuring the dispersion becomes increasingly difficult over time for the power-law network. The reason for this is that the epidemic tends to die out early with a high probability. Thus, the dispersion is estimated on a comparatively small amount of samples. At the same time, the variance of the distribution is comparably high.*
> *This leads to a noticeable amount of noise.*

6) What is the justification for why do the authors have specifically employed the power-law, household, and Watts-Strogatz contact networks? I suggest that the authors make this more explicit in the manuscript.

Finding a single one-fits-all network that captures all relevant scenarios of human connectivity among different populations and age groups and with strict or loose NPIs is hopeless.
On the other hand, it would not be sufficient to find a particular contact network and show that this gives rise to different dynamics than a homogenous population (without motivating its relevance for human-to-human connectivity)

Instead, we considered what are well-known features of human connectivity (like households, small-worldness, super-spreaders) and then we picked three networks that embrace these properties.
The three networks are very different considering their high-level features. However, they all support our claim that the contact structure shapes the dynamics of the epidemics.

We clarified this in the manuscript (248):

> *Apart from the baseline (complete graph), we use specifically these three network models because they are well-studied in literature and very different in their respective global properties. Moreover, they all encode important properties of human-to-human connectivity like hubs (power-law), small-worldness (power-law and WS) and tightly connected household structures.*

7) In line 414, the authors write: …
As a matter of fact, ODE models are not based on "assumptions and abstractions". Instead, one has well-established ODE models, which can give us relevant information about the mitigation of the Covid-19. In this respect, the authors should reformulate such sentence and cite the following references:

The references are indeed very useful to support the relevance of SIR models and we happily include them in our manuscript (line 91 and 107).
In this passage, we wanted to express that all models include certain abstractions.
To emphasize our point, we reformulated the passage as follows (line 440):

> *Naturally, mathematical modeling is based on assumptions and abstractions. However, heterogeneity seems to be particularly vital and excluding it should only be done with great caution. It is particularly difficult to capture population heterogeneity in the widely-used class of ODE models. This is due to their inherent homogeneity assumptions w.r.t. each compartment. Although effects such as overdispersion can be modelled to some extent using this paradigm [90], the complex interplay of varying infectiousness and connectivity remains mostly elusive for such models. Discussing epidemics in terms of population averages may not adequately reflect the complexity of the emerging dynamics.*

8) The authors should state in a more clear-cut way what is new in the present work based on similar publications employing network models for COVID-19, since after the COVID-19 outbreak, an uncountable number of articles have been published employing distinct models aiming to describe the COVID-19 spread.

Our work is in fact a consequence of the myriad of COVID-19 spreading models and we want to highlight important aspects often overlooked. Thus, instead of, e.g., forecasting the trajectory of the epidemic, we take a step back and study the models and their shortcomings and pitfalls.
We highlight this in the introduction (line 8):

> *Literature abounds with new studies describing and forecasting the spread of COVID-19. Instead, we focus on fundamental properties of popular models and the consequences of popular modelling assumptions.*

Review 2

I therefore believe that the authors should support the broad statement made in the title, i.e. that ODE models fail, by showing or explaining why they believe, for example, that their conclusions apply when additional approaches are used in fixing the parameters between the models, including data driven approaches.

We changed the title of the manuscript to "*Heterogeneity matters: Contact structure and individual variation shape epidemic dynamics*".
It is true that time-dependent infection rates are a widely-used mechanism to increase the expressiveness of ODE models. However, we consider time-dependent infection rates to be rather unprincipled as they encode changes in the contact structure as well as changes in the environment.
For instance, in the power-law example, the time-dependent infection rate of the corresponding ODE-model would change drastically and multiple times over the course of the epidemic. However, the underlying contact structure and the NPIs remain the same. Thus, while this makes fitting an ODE to data easier, it does not solve the underlying problem associated with the homogeneity assumption.

Given that ODE approaches relate the compartments as fractions and are therefore independent of the population size, the authors should comment on the choice of N and should argue how their conclusions may vary with this choice. For example, can the discrepancy between the ODE result and the fully connected, homogeneous network be an indication of the error made by the choice of N...

This is a very important point. The reason for using N=1000 was made due to resource constraints (N=1000 still allowed a number of simulation runs, large enough to estimate the stochastic variation in R_t, in reasonable time).
Note that the fully connected graph is, loosely speaking, the graph equivalent to the ODE-model. In Fig. 6, one can clearly see that the two infection curves are very similar to each other already. For increasing N, the two curves become even closer together. We can see this as an indication that we are already at a point where the network size is not dominant for the spreading. There are also sound theoretical reasons to believe that the differences are mainly due to the network structure and not the choice of N. For instance the epidemic threshold [84], and not N, measures how well a network supports the spreading of an epidemic.
Moreover, epidemic spreading on infinite graphs is also well-studied in the literature using various types of mean-field abstractions and it is well-known that different graph topologies can be associated with different outbreak dynamics. Thus, we added (line 291):

> *The number of 1000 nodes was used for practical reasons, however, increasing the network size preserves the qualitative characteristics of the dynamics.*

However, if I read Figs. 7 or 8 correctly, for the power-law case, one can see that 80% of the infected are from between 10 - 20% of the infected nodes after the tenth day or so, …

This interpretation is correct. We wanted to point out that this does not happen immediately on day 0. We rephrased the sentence for clarification.
We changed the passage to (line 397):

> *Regarding the dispersion, we see that none of the considered network structures by itself leads to a dispersion where 80% of the infections are caused by only 15% of the infected nodes (at least not right from the beginning, in the power-law graph this point is approximately reached within a few days).*

We are also very thankful for the detailed minor comments and addressed each of them.
For instance, why we included branching processes or how to compute R_0 for the ODE.

We hope that the changes made to the manuscript appropriately addressed the feedback and concern. In our opinion, the suggested changes greatly improved the clarity of our article.

Kind regards,
Gerrit Großmann
